# Real‐Time Continuous Glucose Monitoring Use and Kidney Function Among People With Diabetes and CKD or At‐Risk for CKD

**DOI:** 10.1002/edm2.70344

**Published:** 2026-10-22

**Authors:** Katia L. Hannah, Christina Chen, Jessica R. Castle, Gregory J. Norman, Rodolfo J. Galindo

**Affiliations:** ^1^ Clinical Affairs Dexcom, Inc. San Diego California USA; ^2^ Medical Affairs Dexcom, Inc. San Diego California USA; ^3^ Division of Endocrinology, Diabetes and Metabolism University of Miami Miller School of Medicine Miami Florida USA

**Keywords:** chronic kidney disease, continuous glucose monitoring, diabetes mellitus, HbA1c

## Abstract

**Introduction:**

Continuous glucose monitoring (CGM) use may benefit people with diabetes and chronic kidney disease (CKD). In this propensity‐matched, retrospective analysis using Truveta‐provided de‐identified EMR data, changes in kidney function and glycemia were compared between cohorts starting real‐time CGM (RT‐CGM) use and CGM non‐users (No CGM).

**Methods:**

Adults with type 1 or type 2 diabetes, treated with any insulin, with estimated glomerular filtration rate (eGFR) of 15–89 mL/min/1.73 m^2^ were included. Changes in eGFR, albumin‐to‐creatinine ratio (ACR), and HbA1c between baseline and 3‐year follow‐up were assessed.

**Results:**

RT‐CGM initiation (*N* = 518 per cohort) was associated with a significantly slower decline in eGFR compared to No CGM. There was no significant difference in change in ACR between the groups (*N* = 138 per cohort). RT‐CGM use (*N* = 461 per cohort) was associated with a significantly greater decrease in HbA1c compared to No CGM.

**Conclusions:**

These findings support RT‐CGM initiation among people with diabetes and CKD or at risk for CKD.

## Graphical abstract

In this retrospective analysis using Truveta de‐identified electronic medical record data, real‐time continuous glucose monitoring (RT‐CGM) initiation (*N* = 518 per cohort) was associated with a significantly slower decline in eGFR compared to no CGM use, supporting RT‐CGM initiation among people with diabetes and chronic kidney disease (CKD) or at risk for CKD.

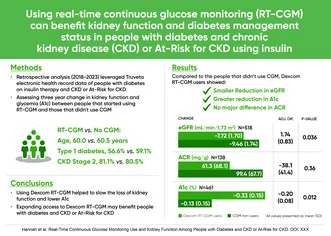

## 1 Introduction

Chronic kidney disease (CKD) is a common complication and comorbidity in people living with diabetes [1]. Glycemic management in people with diabetes and CKD is challenging because of changes in anti‐diabetic medications clearance, higher hypoglycemia risk and poor accuracy of haemoglobin A1c (HbA1c) [2].

A recent consensus report advocated for greater use of continuous glucose monitoring (CGM) and additional research in this population [3]. In this analysis of real‐world electronic health records (EHR), we assessed changes in kidney function and glycemia after starting real‐time CGM (RT‐CGM) use compared to CGM non‐users (No CGM) in people with insulin‐treated diabetes and CKD.

## 2 Methods

This retrospective analysis used de‐identified data from Truveta Data: a U.S. national EHR database representing more than 130 million adults and includes patient demographics, diagnoses, encounters, and medications. Adults with Type 1 or Type 2 diabetes (T1D or T2D), treated with any insulin, with estimated glomerular filtration rate (eGFR) of 15–89 mL/min/1.73 m^2^ (estimated by the creatinine‐based CKD‐EPI formula) were identified. An eGFR value up to 89 mL/min/1.73 m^2^ was allowed to capture individuals at‐risk for CKD who may have developed CKD at the 3‐year follow‐up. Exclusion criteria included prior CGM use, dialysis, kidney transplant, Elixhauser score ≥ 10, liver cirrhosis, pregnancy, or anaemia.

Individuals in the RT‐CGM group initiated a Dexcom G‐series RT‐CGM between 08/01/2018 and 12/31/2023. Index dates in the RT‐CGM group were the first prescription fill for RT‐CGM. One RT‐CGM refill within 6 months from the index date was also required. Individuals in the control group (No CGM) were assigned index dates based on their first outpatient encounter with an eGFR lab order. We assessed changes in eGFR, albumin‐to‐creatinine ratio (ACR), and HbA1c between baseline and 3‐year follow‐up. The baseline value was the closest value to the index date within a −12‐month to +7‐day window. The follow‐up value was the closest value to exactly 3 years post‐index date (within a ±6‐month window).

This analysis consisted of three models; each model estimated the covariate‐adjusted average change from baseline over the 3‐year follow‐up period. The model assessing changes in eGFR required baseline eGFR, baseline HbA1c, and follow‐up eGFR. The model assessing changes in HbA1c required those same values plus a follow‐up HbA1c value. The model assessing changes in ACR required baseline ACR, baseline eGFR, baseline HbA1c, and follow‐up ACR. Propensity‐score matching (1:1) was performed without replacement within each cohort. Multivariable linear regression estimated the association between the initiation of RT‐CGM and changes in outcomes. The groups were propensity‐score matched using the nearest neighbour algorithm with a caliper of 0.2 on age, gender, race/ethnicity, insurance type, diabetes type, baseline eGFR, baseline HbA1c, baseline ACR (albuminuria model only), and use of sodium‐glucose cotransporter 2 inhibitors, incretin mimetics (GLP‐1 and dual GIP/GLP‐1 receptor agonists), or renin‐angiotensin‐aldosterone system inhibition therapies. Characteristics used as covariates were identical to the propensity‐score matching characteristics with the addition of census region and history of hypertension, microvascular complications, and macrovascular complications.

## 3 Results

Baseline characteristics are reported in Table 1. In the model assessing changes in eGFR (*N* = 518 per cohort, median [range] follow‐up of 3.0 [2.5–3.4] years), RT‐CGM initiation was associated with a significantly slower decline in eGFR compared to No CGM after adjusting for covariates (Table 2). In the model assessing changes in ACR (*N* = 138 per cohort, median follow‐up 2.9 [2.5–3.4] years), the change in ACR was not significantly different between the groups. In the model assessing changes in HbA1c (*N* = 461 per cohort, median follow‐up 3.0 [2.5–3.4] years), RT‐CGM use was associated with a significantly greater decrease in HbA1c compared to No CGM.

**TABLE 1:** Baseline characteristics of the matched cohort.

Characteristic	RT‐CGM	No CGM
*N*	518	518
Age at index, mean (SD)	60.0 (12.7)	60.5 (12.8)
Age		
18–30	9 (1.7)	9 (1.7)
30–44	55 (10.6)	54 (10.4)
45–54	101 (19.5)	97 (18.7)
55–64	160 (30.9)	142 (27.4)
65+	193 (37.3)	216 (41.7)
Female	271 (52.3)	271 (52.3)
Race/Ethnicity		
Asian American	3 (0.6)	2 (0.4)
Black or African American	58 (11.2)	65 (12.5)
Hispanic	15 (2.9)	15 (2.9)
Other race	15 (2.9)	14 (2.7)
White	427 (82.4)	422 (81.5)
US census region		
Midwest	101 (19.5)	110 (21.2)
Northeast	25 (4.8)	20 (3.9)
South	103 (19.9)	137 (26.4)
Unknown	211 (40.7)	202 (39.0)
West	78 (15.1)	49 (9.4)
Insurance type		
Public (Medicare/Medicaid)	115 (22.2)	113 (21.8)
Private	403 (77.8)	405 (78.2)
Diabetes type^a^		
Type 1	293 (56.6)	306 (59.1)
Type 2	225 (43.4)	212 (40.9)
eGFR (mL/min/1.73 m^2^), mean (SD)	71.2 (14.6)	70.9 (14.2)
eGFR group		
G2	420 (81.1)	417 (80.5)
G3a	63 (12.2)	71 (13.7)
G3b	30 (5.8)	24 (4.6)
G4	5 (1.0)	6 (1.2)
ACR (mg/g)^b^, mean (SD)	115 (306)	116 (473)
HbA1c (%), mean (SD)	8.0 (1.5)	8.0 (1.8)
Microvascular complications^c^	114 (22.0)	77 (14.9)
Macrovascular complications^c^	203 (39.2)	202 (39.0)
Hypertension^c^	380 (73.4)	400 (77.2)
RAASi therapy	363 (70.1)	372 (71.8)
Incretin mimetic therapy	110 (21.2)	93 (17.8)
SGLT‐2 inhibitor therapy	72 (13.9)	74 (14.3)

Abbreviations: ACR, albumin‐to‐creatinine ratio; CGM, continuous glucose monitoring; eGFR, estimated glomerular filtration rate; RAASi, renin‐angiotensin‐aldosterone system inhibition; SD, standard deviation; SGLT‐2, sodium‐glucose cotransporter 2.

^a^ Determined using the modified Klompas algorithm for detecting and classifying T1D vs. T2D using EHR data where diabetes type was determined by the diagnosis code most often observed (i.e., the majority diagnosis code).

^b^ *N* = 138.

^c^ Prevalence of complications was determined from diagnosis codes. Microvascular complications included neuropathy and retinopathy (nephropathy was excluded); macrovascular complications included myocardial infarction, stroke, and other atherosclerotic cardiovascular disease complications. Data presented as *n* (%) unless otherwise indicated.

**TABLE 2:** Summary of eGFR, ACR, and HbA1c outcomes from baseline to 3‐year follow‐up.

	Change from baseline	Adjusted change	*p*
**eGFR, mL/min/1.73 m** ^**2**^			
RT‐CGM	−7.72 (1.70)	1.74 (0.83)	0.04
No CGM	−9.46 (1.74)		
**ACR, mg/g**			
RT‐CGM	61.3 (68.1)	−38.1 (41.4)	0.36
No CGM	99.4 (67.7)		
**HbA1c, %**			
RT‐CGM	−0.33 (0.15)	−0.20 (0.08)	0.01
No CGM	−0.13 (0.15)		

*Note:* Data presented as mean (SE).

## 4 Discussion

In this propensity‐matched, retrospective analysis, RT‐CGM initiation among people with insulin‐treated diabetes and CKD or at risk for CKD was associated with statistically significant improvements in the rate of kidney function decline and HbA1c change during a follow‐up of 3 years compared to people not using CGM.

Extensive evidence supports the use of CGM in with Type 1 and Type 2 diabetes [4], however, there is less evidence among people with CKD or at‐risk for CKD. A recent consensus report advocated for additional research in this population to determine “the effects of CGM on reducing hypoglycemia/hyperglycemia, CKD progression, and cardio‐kidney‐metabolic risk” [3]. In the present study, we demonstrated that people with diabetes and CKD Stage 2–4, who started using RT‐CGM, had a slower decline in kidney function.

Persistent chronic hyperglycemia is a key driver for development and worsening of diabetic kidney disease [5], and several studies support that improving HbA1c helps delay CKD progression [1]. Our study adds to this evidence and found that RT‐CGM use was associated with HbA1c improvements. We hypothesize that RT‐CGM may be facilitating lifestyle modifications, medication adherence, and/or medication optimization that support glucose management [6]. In addition, a recent study in people with T2D and CKD found that CGM initiation was associated with 17.0% and 18.2% reductions in rates of hypoglycemia and hyperglycemia crises, respectively [7]. While prior clinical trials have reported greater improvements in HbA1c following CGM initiation than what was observed in this analysis [8], HbA1c measurements have demonstrated reduced accuracy in people with advanced CKD, including those treated with dialysis [3]. However, despite this limitation of HbA1c, it is widely used in clinical practice.

Strengths of this study include its longitudinal design with a 3‐year follow‐up and use of a nationally representative database of integrated health systems. The study is limited by the relatively low number of individuals with advanced kidney disease. In addition, this analysis did not exclude acute kidney injury events. Adherence to RT‐CGM was also not assessed beyond the requirement of at least one refill within 6 months of the index date. The extent of propensity score matching in the eGFR and HbA1c models was also limited by the available data and did not include baseline ACR (a potential confounder associated with CGM initiation). It was also not possible to include some factors that impact metabolic and renal outcomes, such as indicators of medication intensification, socioeconomic status, education level, or care quality like type of provider or rate of healthcare utilization. In addition, a longer follow‐up time (≥ 5 years) may allow for greater outcome changes. Future analyses should conduct sensitivity analyses to address the possibility of residual confounding. Because of these limitations, a causal relationship between CGM use and the observed outcomes cannot be determined.

Overall, our results suggest that RT‐CGM initiation could be considered among people with diabetes and CKD or at risk for CKD. Given the low accuracy of HbA1c in this population, future prospective studies should assess whether improvements in CGM metrics, such as time‐in‐range, mean glucose, or glycemic variability, correlate with delayed CKD progression.

## Author Contributions

**Katia L. Hannah:** conceptualization, data curation, formal analysis, methodology, project administration, writing – original draft, writing – review and editing. **Christina Chen:** conceptualization, methodology, writing – original draft, writing – review and editing. **Jessica R. Castle:** conceptualization, methodology, supervision, writing – review and editing. **Gregory J. Norman:** conceptualization, methodology, project administration, supervision, writing – original draft, writing – review and editing. **Rodolfo J. Galindo:** methodology, supervision, writing – original draft, writing – review and editing.

## Funding

Dexcom Inc. funded the development of this article.

## Conflicts of Interest

K.L.H., C.C., J.R.C., and G.J.N. are employees of Dexcom Inc. R.J.G. is supported in part by grants from the National Institute of Diabetes and Digestive and Kidney Diseases (NIDDK) of the National Institute of Health (NIH) under award numbers P30DK111024, 1K23DK123384, and 1R03DK138255. R.J.G. received research support from Novo Nordisk, Eli Lilly, and Dexcom, and consulting/advisory from Abbott Diabetes, Bayer, Boehringer, Dexcom, Eli Lilly, and Novo Nordisk, and Medtronic.

## Data Availability

Research data are not shared.
